# Malignant atypical cell in urine cytology: a diagnostic dilemma

**DOI:** 10.1186/1742-6413-3-28

**Published:** 2006-12-15

**Authors:** Alka Bhatia, Pranab Dey, Nandita Kakkar, Radhika Srinivasan, Raje Nijhawan

**Affiliations:** 1 Department of cytology, Post Graduate Institute of Medical Education and Research, Chandigarh, India; 2 Department of Histopathology, Post Graduate Institute of Medical Education and Research, Chandigarh, India

## Abstract

**Aims:**

The aim of this study was to find out the characteristic morphology of malignant atypical cells which were missed on routine cytology of urine.

**Materials and methods:**

In this retrospective study, we examined detailed cytomorphology of 18 cases of atypical urinary cytology which were missed on routine examination and were further proved on histopathology as transitional cell carcinoma (TCC) of bladder. The cytological features of these cases were compared with 10 cases of benign urine samples.

**Results:**

There were 11 cases of high grade TCC and 7 cases of low grade TCC on histopathology of the atypical urine samples. Necrosis in the background and necrosed papillae were mostly seen in malignant atypical cells. The comet cells and cells with India ink nuclei (single cells with deep black structure-less nuclei) were only observed in malignant atypical cells. The most consistent features in malignant atypical cells were: i) high nuclear and cytoplasmic (N/C) ratio ii) nuclear pleomorphism iii) nuclear margin irregularity iv) hyperchromasia and v) chromatin abnormalities

**Conclusion:**

The present study emphasizes that nuclear features such as high N/C ratio, hyperchromasia and chromatin abnormalities are particularly useful for assessing the malignant atypical cells. Other cytological features such as comet cells and cells with India ink nuclei are also helpful for diagnosis but have limited value because they are less frequently seen.

## Background

Urine cytology for screening of transitional cell carcinoma (TCC) has been used for long time. Despite the advent of several newer techniques for screening and diagnosis of urothelial malignancies, cytomorphology still remains an important tool [1]. "Atypical cells" in urine have been recognized and studied time and again [2,3]. The accurate interpretation of the character of "Atypical cell" in urine is a major challenge for cytopathologists. In this present paper we analyzed the characteristic morphology of malignant atypical cells which were missed on routine cytology of urine.

## Materials and methods

A total of 2997 voided urine samples received in our laboratory over a period of one and half year (January 2005-June 2006) were studied. A total of 58 urine samples reported as positive for atypical cells were initially selected. Out of these cases, the histological correlation was available in 54 samples (18 cases, three consecutive samples) and these were finally selected for the present study. Out of these, 11 cases were reported as high grade papillary urothelial carcinoma and 7 cases were reported as low grade papillary urothelial carcinoma on histology according to International society of urological pathology classification system [4].

Also cytomorphology of 30 samples (10 cases) of non-neoplastic urinary samples were compared with these atypical urinary samples. These cases were negative for malignancy on cystoscopy and follow up history.

Two Papanicolaou stained smears were examined for each sample. The slides were seen by two independent observers (AB and PD).

The detailed cytological features such as cellularity, cell clustering, papillae, comet cells, India-ink cells, necrosis, apoptosis, nucleo-cytoplasmic ratio, cytoplasmic detail, nuclear pleomorphism, nuclear margin, hyperchromasia, and chromatin abnormalities were observed.

## Results

**Figure 1:**
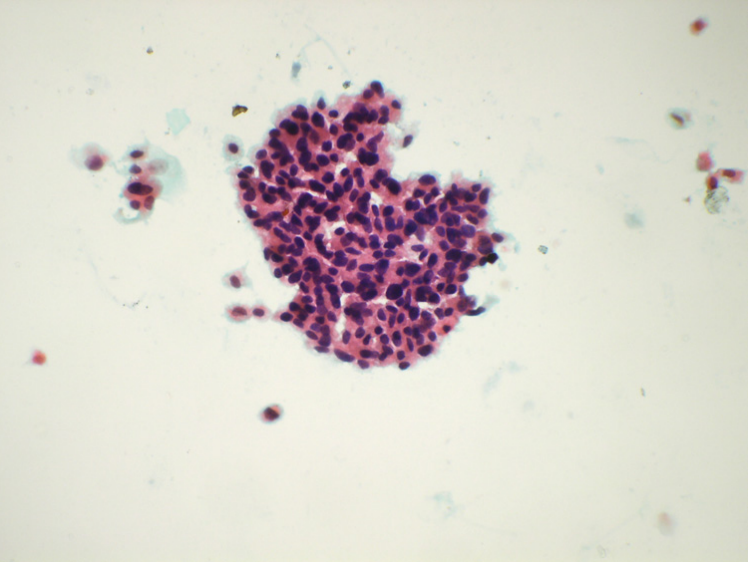
Papillary cluster of malignant atypical cells (Papanicolaou's stain × 550).

**Figure 2:**
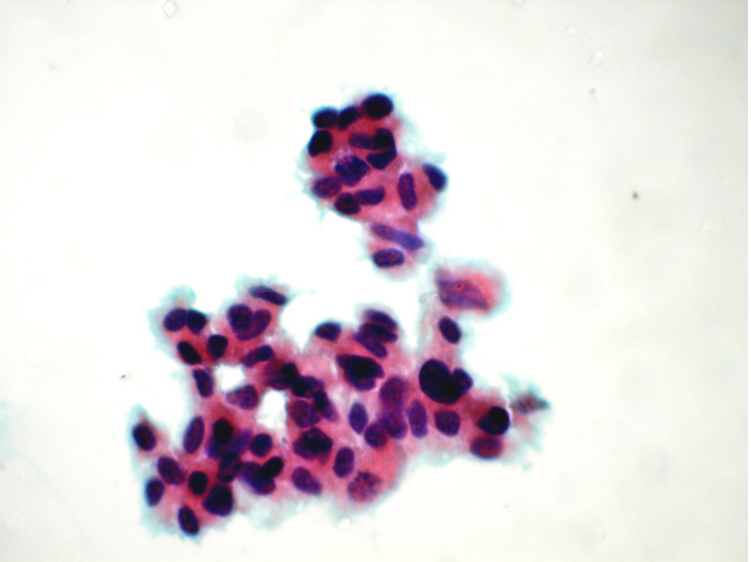
Cluster of malignant atypical cell showing nuclear pleomorphism (Papanicolaou's stain ×1375).

**Figure 3:**
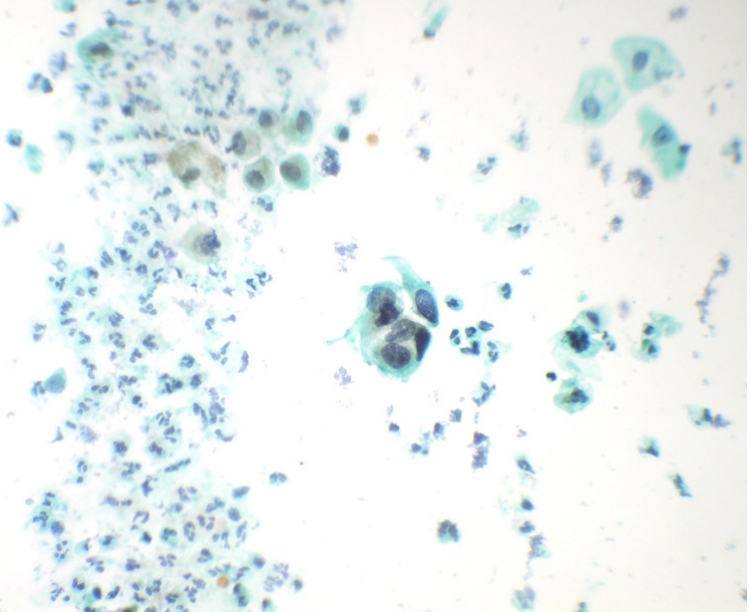
Smear shows tiny cluster of malignant atypical cells. (Papanicolaou's stain × 550).

**Figure 4:**
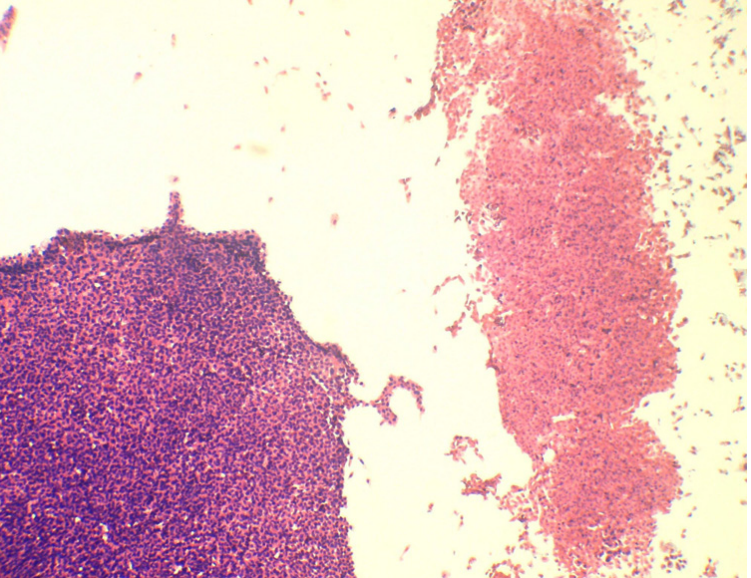
A large cell cluster of cells show necrosis. Part of a viable cluster is also identified (Papanicolaou's stain × 280).

**Figure 5:**
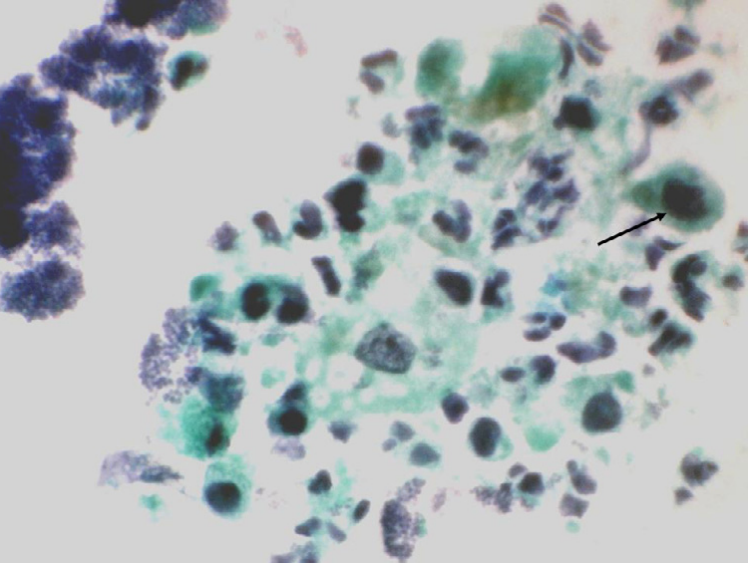
Comet cells and cells with India ink nuclei (arrow) seen in a necrotic background (Papanicolaou's stain × 550).

There were a total of 18 malignant atypical cases in this study. The cytological features are highlighted in table 1 and 2. Most of our atypical cases had increased cellularity (11). In two of the cases, the inflammatory cells were obscuring the field and atypical cells were scattered singly or in tiny clusters in between them. Cellularity was low in three cases due to a haemorrhagic background. The cell clustering was observed in 11 cases however in most of them the clusters were few (figures 1, 2, and figure 3). Only one case with high grade papillary urothelial carcinoma showed many large clusters (figure 4). The comet cells and cells with India ink nuclei (single cells with deep black structure-less nuclei) were observed in total seven and five cases respectively (figure 5). Necrosis, both in background as well as necrosed papillae (figure 4) were observed in 50% of the cases and these were commonly observed in high grade papillary urothelial carcinoma.

**Table 1:** Detailed general cytological features in smears containing malignant atypical cells

Serial number	Cellularity	Clusters	Papillae	Comet cell	India ink	Necrosis	Apoptosis	Histological Grade
1	+++	-	-	+	+	-	+	H
2	+	+	+	-	-	-	-	H
3	+	+	-	+	+	+	+	H
4	++	+++	-	+	+	++	+	H
5	+++	-	-	+	+	+++	+	H
6	+++	-	-	-	-	+++	-	H
7	+++	++	++	-	-	+	++	H
8	++	+	-	-	-	-	+	H
9	++	+	-	-	+	-	-	H
10	++	+	-	+	-	+	-	H
11	+	-	-	+	-	+	-	H
12	+++	+	-	+	-	++	+	L
13	+	-	-	-	-	-	-	L
14	+	-	-	-	-	-	-	L
15	+++	++	+	-	-	-	+	L
16	+	+	-	-	-	-	+	L
17	++	+	-	-	-	++	-	L
18	+	-	-	-	-			L

+ = mild/few, ++ = moderate, +++ = severe/many, H = High, L = Low

**Table 2:** Detailed cytological features in malignant atypical cells

Serial number	Shape & size	N/C ratio	Cytoplasm	Nuclear pleomorphism	Irregular Nuclear margin	Hyperchromasia	Chromatin	HPR
1	A	↑	angulated	++	+	+	coarse	H
2	A	↑		+	+	+	homogenous	H
3	A	↑		+++	+	+	coarse	H
4	A	↑	angulated pale	++	+	+	coarse	H
5	A	N	pale	+	+	-	homogenous	H
6	A	↑		++	+	+	coarse	H
7	A	↑		++	+	+	homogenous	H
8	A	↑	angulated	+	+	++	homogenous	H
9	A	↑	-	++	+	+	coarse	H
10	A	↑	-	+	+	+	coarse	H
11	A	N	-	-	-	+	homogenous	H
12	A	↑	pale	+	+	+++	homogenous	L
13	N	↑		+	-	+	homogenous	L
14	N	↑		+	-	+	homogenous	L
15	A	↑	pale	++	+	+	homogenous	L
16	A	↑	angulated pale	+	+	+	coarse	L
17	N	↑		+	+	+	homogenous	L
18	A	N	-	+	+	+	coarse	L

+ = mild/few, ++ = moderate, +++ = severe/many, A = altered, N = normal, N/C = nuclear/cytoplasmic, ↑ = High, HPR = Histopathology report

**Figure 6:**
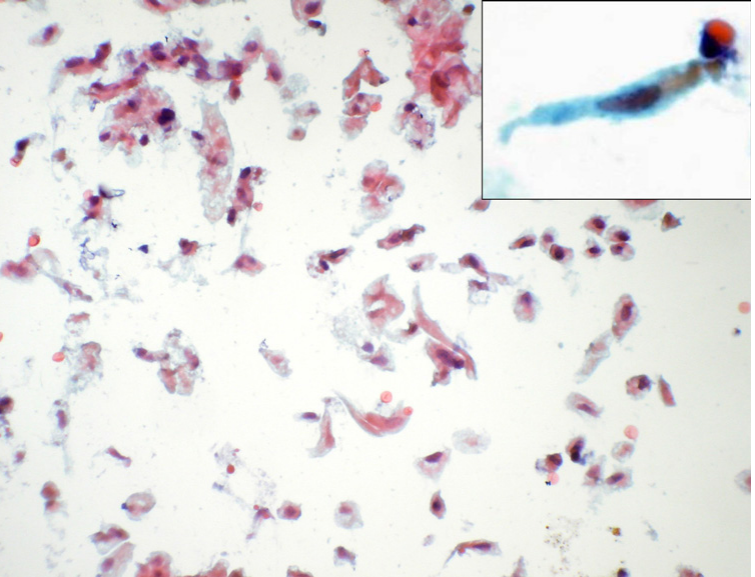
Singly scattered spindle cells seen in some of the smears (Papanicolaou stain × 280). Inset shows a high power view of the same.

Apoptosis is the active process of programmed death of cells without provoking any inflammatory responses. The cell death by apoptotic process was detected on the smear without much difficulty. The individual apoptotic cells were present singly. The cells were round to oval, smaller in size with dense cytoplasm. In addition the apoptotic cells showed dark, pyknotic nuclei of variable sizes. Apoptosis (9 cases) was more commonly observed in high grade tumors although it was also noted in some low grade tumors (3) Spindle shaped cells were identified in 3 cases (figure 6). One case showed significant degenerative changes in the smear.

**Figure 7:**
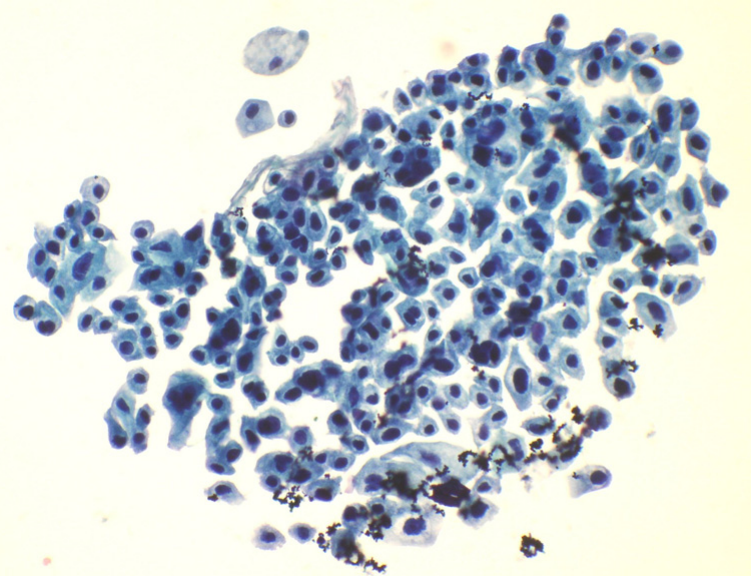
Nuclear & cytoplasmic features in atypical cells: marked pleomorphism and high nuclear and cytoplasmic ratio with pale and angulated cytoplasm (Papanicolaou's stain × 550).

**Figure 8:**
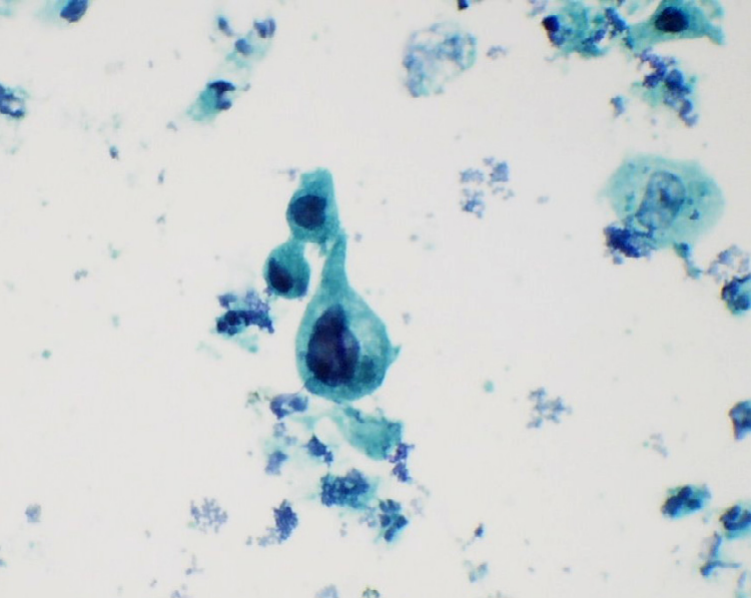
Atypical cells with irregular nuclear margins and nuclear hyperchromasia (Papanicolaou's stain × 1375).

In majority of the cases (15) the atypical cells showed alteration in shape and size (table 2). The cytoplasmic changes in the form of paleness and angulations were observed in 7 of our patients (figure 7). Four most consistently observed features in atypical cells were: i) high nuclear: cytoplasmic ratio (83.3%) ii) nuclear pleomorphism (94.4%) iii) nuclear margin irregularity (88.9%) and iv) hyperchromasia (94.4%) (figures 7, 8). These features were present irrespective of the grade of tumors. Chromatin abnormalities either in the form of coarse clumping or a homogenous pattern was also noted (100%).

In contrast to the above, the non-neoplastic samples were characterized by a low cellularity. Cell clusters were tiny and cells were in groups of 4–5, in contrast from neoplastic clusters which were small to large (5–40 cells). Comet, India-ink and apoptotic cells were not observed in any of the non-neoplastic samples. Necrosis was focal, however diffuse background necrosis was seen in two cases with accompanying heavy inflammation. In non neoplastic lesions, occasional mildly pleomorphic cells were observed, however cytoplasmic changes and the other nuclear changes such as high nuclear: cytoplasmic (N/C) ratio, hyperchromasia and abnormal chromatin pattern were consistently absent (table 3).

**Table 3:** Comparison of cytological features of Malignant atypical and non-neoplastic smears

Cytomorphologic feature	Atypical malignant n = 18	Non-neoplastic n = 10
Increased cellularity	11 (61.1%)	1 (10%)
Clustering	11 (61.1%)	3(30%)
Papillae	3(16.67%)	0
Comet cells	7 (38.9%)	0
India-ink cells	5 (27.78%)	0
Necrosis	9 (50%)	4(40%)
Apoptosis	9 (50%)	0
Shape and size changes	15 (83.33%)	7(70%)
High nuclear/cytoplasmic ratio	15 (83.33%)	0
Cytoplasmic alterations	8 (44.44%)	0
Nuclear pleomorphism	17(94.44%)	6(60%)
Irregular margin	16 (88.9%)	2(20%)
Hyperchromasia	17 (94.44%)	0
Chromatin abnormalities	18 (100%)	0

## Discussion

Urine cytology is an important non-invasive technique for screening, diagnosis and follow-up of patients with urinary bladder cancer. Despite the advancement in laboratory equipment for processing of the specimens and availability of ancillary techniques for diagnosis, difficulties are still encountered in some cases [5]. In these cases, the recognition of exact nature of the "atypical cells" in urine is really difficult.

Increased cellularity and cell clustering was previously mentioned as helpful in diagnosis of malignancy [2], however in our study we noted these features in many benign urine samples also. This may be due to the fact that exfoliation of transitional cells can be caused by a variety of diseases like lithiasis, instrumentation, inflammatory diseases and benign prostatic hyperplasia [6]. In the present study papillae, India ink nuclei and comet cells were noted exclusively in malignant cases. However these features had low frequency for diagnosis of malignancy. The necrosis was seen exclusively in malignancies in previous studies [1]. We noted necrosis in 40% of our non-neoplastic smears also. Apoptosis however was observed only in malignant atypical smears of high grade TCC in histology.

Alterations in nuclear morphology were most consistently observed in the malignant atypical cells. High N/C ratio, hyperchromasia, chromatin alterations, nuclear pleomorphism and irregular nuclear margins were most frequently encountered features in malignant atypical cells. Out of these, the first three features were seen only in malignant atypical cells. Raab et al [1] in their study on low grade papillary urothelial neoplasms mentioned that high N/C ratio, irregular borders and cytoplasmic homogeneity are the key diagnostic criteria. In our study, the nuclear pleomorphism and margin irregularities were seen more frequently in malignant atypical smears and rarely observed in few benign cases.

Cytoplasmic changes in the form of angulations and paleness were seen in malignant atypical cases. However, the frequency of these features was low.

Spindle shaped cells were seen in 3 of our cases and the histopathology report in all 3 of them was high grade papillary urothelial carcinoma. Previous studies have also mentioned a similar association of spindle cells with TCC [2,5].

The major challenge for the cytopathologist lies in distinguishing between a low grade transitional cell carcinoma and reactive atypia due to stone, viral infections, instrumentation and chemotherapy [7]. In our study, the above mentioned nuclear features combined with a relevant clinical history were of great help in differential diagnosis. Maier et al [8] also found better results when more clinical information was made available to the pathologist and standard protocol was used for collection of urine specimens.

There was considerable overlap in the features observed in low grade and high grade tumors. However certain features like necrosis, apoptosis, nuclear and chromatin changes were more frequent or more severe in high grade lesions.

The various ancillary techniques could be combined with cytomorphology to increase the diagnostic accuracy include monoclonal antibodies to cytokeratin20 [9], flow cytometry of bladder irrigation specimens [10], cytogenetics [11] and nuclear morphometry [12].

In brief, we noted that the presence of India ink cell, comet cells and apoptotic cells were solely present in malignancy related atypia. Along with these, the nuclear features like high N/C ratio, hyperchromasia and chromatin abnormalities are particularly useful in diagnosis of malignancy. The newer ancillary techniques could be used along with routine microscopic examination to increase the diagnostic accuracy.

## Competing interests

The author(s) declare that they have no competing interests.

## Authors' contributions

AB: Acquisition of data and drafting the manuscript

PD: 1) Conception, design, analysis and interpretation of data and 2) final approval of the version to be published.

NK, SR, RN: Analysis and interpretation of data
